# The influence of age on greater sciatic notch morphology: testing the Walker method in an Australian population

**DOI:** 10.1007/s00414-023-02988-1

**Published:** 2023-04-13

**Authors:** Angel DesMarais, Zuzana Obertova, Daniel Franklin

**Affiliations:** Centre for Forensic Anthropology, School of Social Sciences, The University of Western Australia, 35 Stirling Hwy, Crawley, Western Australia Australia

**Keywords:** Forensic anthropology, Skeletal sex, Pelvis, Age, Morphology

## Abstract

Sex estimation is an integral aspect of a forensic biological profile. The pelvis, being the most dimorphic part of the skeleton, has been studied in considerable detail relative to morphological and metric variation. However, empirical data on the effect of age on pelvic morphology relative to sex-specific morphological variation is limited, especially in regard to the estimation of skeletal sex. This study assesses whether there are age-related differences in the distribution of the Walker (2005) morphological scores for the greater sciatic notch (GSN) in an Australian population. Three-dimensional volumetric reconstructions derived from multi-detector computed tomography (MDCT) scans of 567 pelves of 258 females and 309 males aged 18 to 96 years were scored following Walker (2005). Differences in score distributions and means by sex and age group were tested using Pearson’s chi-squared test and ANOVA, respectively. The accuracy of sex estimates derived from logistic regression equations was explored using leave-one-out cross-validation. Significant differences were found in score distribution and means among age groups in females, but not in males. There was a tendency toward higher scores in older females. The overall sex estimation accuracy was 87.5%. When comparing age groups 18–49 and 70 + years, estimation accuracy decreased in females (99% vs. 91%), while the opposite was found for males (79% vs. 87%). These findings suggest that age affects GSN morphology. Higher mean scores in older females imply that, on average, the GSN becomes narrower with increasing age. It is thus recommended due consideration of estimated age when assessing sex based on the GSN in unidentified human remains.

## Introduction

Sex estimation in the adult skeleton can be accomplished by examining morphological sex-linked trait differences. Sexual dimorphism (characteristics that differentiate males from females) is an expression of the population represented [6, 7, 21]. Published literature attests that the application of standards developed on populations not reflective of the individual referred for assessment leads to potential misclassification in the form of reduced predictive accuracy and/or an increased sex bias [2, 9, 17–20].

Sexually dimorphic characteristics expressed by size, shape, and functional differences between males and females are largely triggered by hormonal responses beginning at the onset of puberty. The degree to which the traits are expressed enables the examiner to quantify the probability of one sex relative to the other [1, 29, 30]. Factors such as age and biomechanical loading can influence the expression of sexually dimorphic characteristics, potentially resulting in inaccurate skeletal sex estimation to the detriment of establishing an identification.

Hormonal changes related to age are known to affect bone morphology [15, 40]. As hormone production starts decreasing around the age of 30 years, bone density slowly begins to diminish in both males and females [15, 40]. Conversely, as age advances, some skeletal regions become more robust with cumulative muscle use, or due to increased bone formation to counteract bone loss or other pathological changes in the skeleton [4, 5]. In the pelvis, a narrowing of the subpubic angle in individuals of advanced age has been reported [36], which may result in inaccurate sex estimation, particularly in elderly females [23, 28, 35, 36, 41].

Walker [42] observed change in greater sciatic notch (GSN) morphology toward the more masculine score distribution of older females. In addition, he noted that younger individuals were associated with a wider GSN than individuals aged 50 + years. He attributed those findings as an artifact of the age structure of his sample populations, i.e., individuals with wider sciatic notches had a greater probability of early death, as the St. Bride’s (English) collection had a larger proportion of older adults (mean age at death, 55.9 years) than the US Hamann-Todd and Terry collections (mean age at death, 50.0 years) [42].

In support of Walker’s original work, additional studies have reported that sex-specific variances in the morphology of the GSN are due to secular changes and population specificity rather than age. Kilmer and Garvin’s [24] study of non-comtemporary populations investigated the applicability of GSN shape outline and discriminant function analysis for sex estimation. Using elliptical Fourier analysis of 2D outlines, the authors demonstrated that the GSN is influenced by population variation, without significant effect of age on its morphology. Additionally, Veleminska et al. [39] examined the accuracy of sex estimation using geometric morphometrics (GMM) of the GSN in Euroamerican and Hispanic populations. These authors suggest that the observed differences in sexual dimorphism of the GSN were due to variability in male morphology and possible population specificity, rather than age.

Conversely, Biwasaka et al. [8] observed that females expressed age-related deepening of the GSN in their analysis of pelvic morphology using a GMM approach. Similar to Biwasaka et al. [8], Byram et al. (2020) also attributed morphological changes in GSN width and height to age using elliptical Fourier analysis. To date, however, there is no research investigating the application of the Walker method relative to the potential interaction of age and sexual dimorphism in the morphology of the GSN.

The aim of the present study is, therefore, to assess the expression of GSN morphology relative to sex following the Walker [42] method in different age groups in a contemporary Australian population. The accuracy of sex estimation and the effect of age is also statistically quantified.

## Materials and methods

### Materials

The present study examined high-resolution multi-detector computed tomography (MDCT) scans of the pelvic region, acquired from Picture Archiving and Communication Systems (PACS) databases, representing participating hospitals across the country. While there are no protocols informing slice resolution [22], it has been reported that utilization of scans > 1 mm may prohibit small details (e.g., sutures, ossification centers, symphyseal surfaces) from being visualized or assessed accurately; scan resolution ≤ 1 mm is recommended for anthropological research [22, 31, 38]. Therefore, as part of the inclusion criteria, only scans with an average of ≤ 1 mm were selected. The sample comprised 309 males and 258 females, aged between 18 and 96 years (mean age = 53 years); see Table 1.

**Table 1:** Age and sex distribution of the Australian sample

Age group (years)	Male	Female	Total
18–29	54	41	95
30–39	28	36	64
40–49	49	43	92
50–59	67	43	110
60–69	56	28	84
70–79	27	29	56
80 +	28	38	66
Total	309	258	567

The scans were reconstructed using volumetric rendering in *OsiriX MD®*. Volumetric rendering was selected over multiplanar for easier and more reliable method application. Volumetric rendering as an accurate reconstruction has been verified in previous studies [11, 14]. Scans presenting obvious congenital or acquired pelvic pathology (e.g., serious fractures) were duly excluded. The scans were anonymized, and only age and sex information has been retained. Research ethics approval was granted by the Human Ethics Office of Research Enterprise of the University of Western Australia (Project No: RA/4/20/5473), and the Human Ethics Committee of the Research of the Northern Territory Department of Health and Menzies School of Health Research (Project No: HREC-2017–2879).

### Methods

**Fig. 1:**
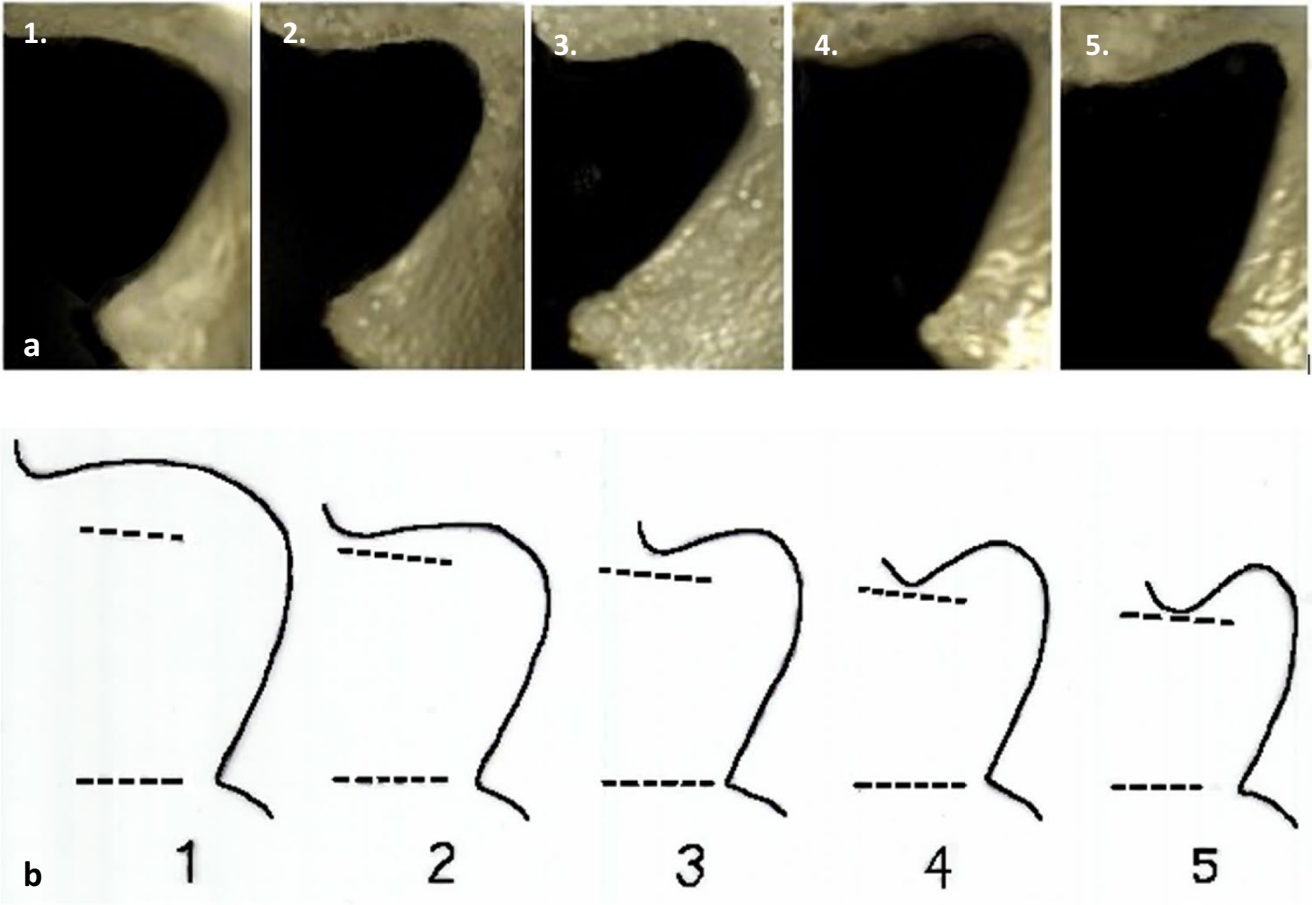
Visualization of Walker [42] method scores. **a** 3-D images representing Walker’s trait scores 1–5 in 3-D volumetric MDCT scans. **b** Transparency of Walker’s illustration depicting trait scores

The Walker [42] method was used to assess the morphology of the greater sciatic notch. The procedure for the method instructs the observer to “hold the *os coxae* about six inches above (the diagram)… align the straight anterior portion of the notch that terminates in the ischial spine with the anterior portions of the sciatic notches illustrated in the diagrams. While holding the bone in this way, move it from diagram to diagram to decide the closest match” [42], p. 386). Consequently, due to the utilization of MDCT scanned images in this study, an adaptation of the method was required. A transparency with an array of the original illustrations of each of the five expressions was superimposed over the appropriately scaled and orientated CT image of the GSN. The illustration that most closely matched the morphology of the GSN being assessed was used to assign a score, as per the original instructions. The assessor was blinded to the age and sex of the scan. Figure 1 shows the greater sciatic notch morphology according to Walker’s method compared to the same stages as reconstructed and visualized from the three-dimensional MDCT scans.

Prior to primary data collection, an assessment of intra-observer precision was performed and bilateral asymmetry assessed. Cohen’s kappa was used to test repeat scoring assignation of greater sciatic notch morphology following Walker [42]. A total of 26 pelvic MDCT scans were selected according to an even sex and age distribution (13 males and females; 25 to 57 years of age). Two repeat assessments were performed, with a 1-week interval between re-assessment. Kappa values were then calculated for both the right and left sides and interpreted according to Landis and Koch [27].

Bilateral symmetry was evaluated to support left-side analysis of samples for this study. Bilateral symmetry of paired morphological traits is a correspondence in size, shape, and relative position of one side to the opposite side [13]. Significant deviation, referred to as bilateral asymmetry, may affect morphoscopic and morphometric analysis by distorting, delaying, or speeding up the skeletal markers used for biological estimations [13, 32]. Multiple factors can influence the prevalence and expression of asymmetry (e.g., genetic, environmental perturbations, handedness, biomechanical, etc.) [26, 34]. Recent research into the bilateral asymmetry of the human pelvis has found few variables resulting in significant asymmetry [26]. To test for bilateral symmetry, Wilcoxon signed-rank test was used to compare the scores for left and right GSN of the same individual to assess bilateral symmetry for the total sample. As there was no significant bilateral asymmetry detected (see below), only the left *os coxa* was used for this study.

Descriptive statistics are used to quantify mean and score distribution of male and female morphologies by age according to three categories: 18–49, 50–69, and 70 + years. The youngest age category was capped at 49 years because 50 years is the mean age of menopause in Australian women [3]. Analysis of variance (ANOVA) was applied to test the differences in means among the age groups for each sex separately. Pearson chi-squared was used to determine the statistical significance for differences between score distributions among age groups for each sex.

Population-specific logistic regression formulae, with sex (0, male; 1, female) as the outcome variable, were created for the total sample and for each age group separately. Two more equations for the total sample also used age and age group as a confounding variable, respectively. Classification accuracies were derived from a leave-one-out cross validation. Sex bias was calculated for the total sample and for each age group by subtracting the female from the male classification accuracy. Negative sex bias values indicate a higher misclassification in males. Statistical analysis was performed using *Microsoft® Office Excel, IBM SPSS Statistics* (v. 27.0), and *RStudio* (v. 1.4.1717) [33].

## Results

Intra-observer agreement was substantial [27] with a mean kappa value of 0.796 (0.743 left and 0.849 right). The Wilcoxon signed-rank test showed that there was no difference between the distribution of scores from the left and right greater sciatic notches of the same individual (*z* =  − 0.577, *p* = 0.5637). Consequently, no significant bilateral asymmetry for the total sample was detected, and only data derived from left *os coxae* are subsequently analyzed.

**Fig. 2:**
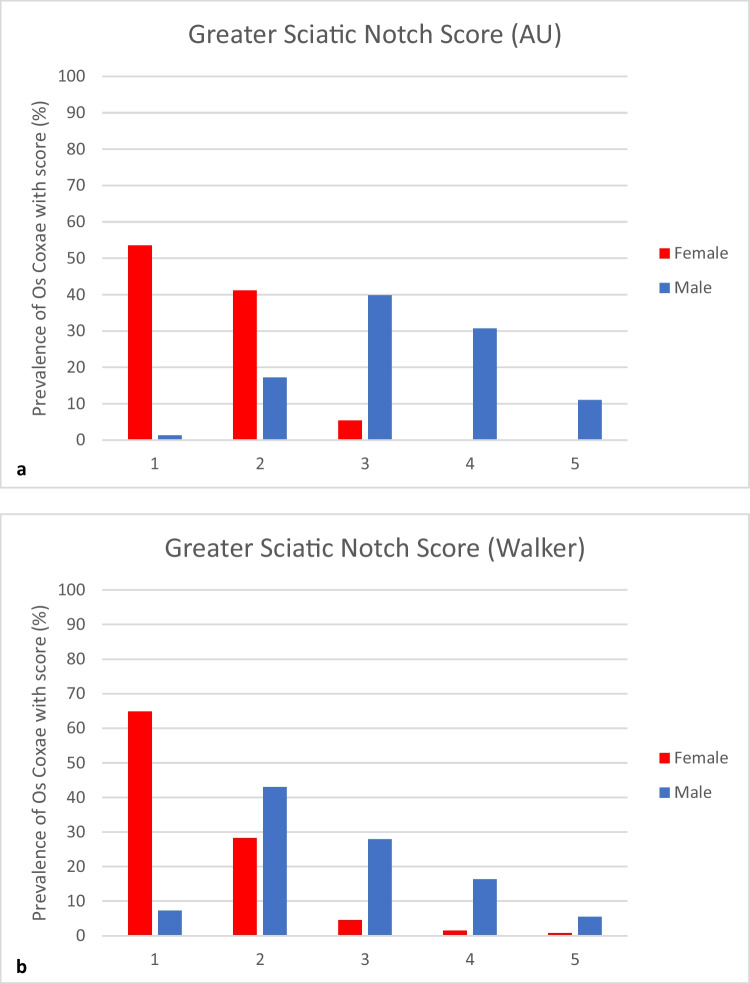
Comparison of greater sciatic notch score distribution for **a** Australian (AU) values  following the Walker [42] method,**b** Walker [42] published values

Figure 2a shows the overall distribution of male and female GSN scores in the Australian sample, while Fig. 2b depicts the distribution of GSN scores reported by Walker [42]. In the Australian sample, 94.6% of females were assigned a score of 1 or 2. The maximum score assigned to females was 3. A similar distribution of scores 1 and 2 was found in the original study by Walker however, 2.3% of his sample was assigned scores of 4 and 5. In comparison, 41.7% of Australian males are scored 4 or 5, and 18.5% a 1 or 2. Walker reported that 21.8% of males had a score of 4 or 5, and 50.3% a score of 1 or 2.

**Fig. 3:**
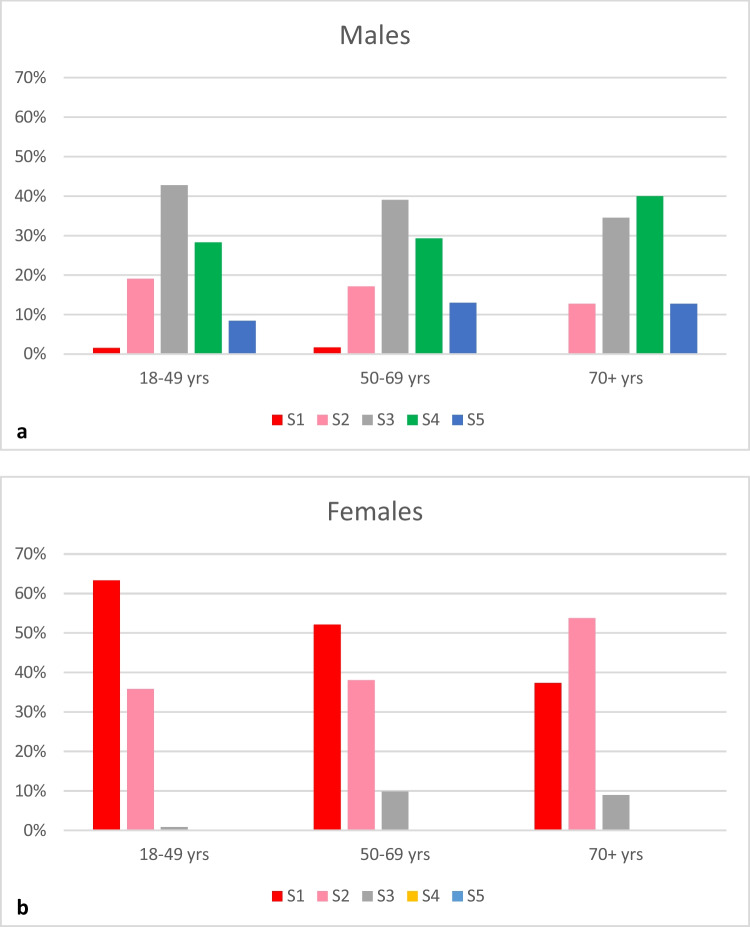
Distribution of greater sciatic notch scores within age groups: **a** Australian males, **b** Australian females

After dividing the Australian sample into age groups, the mean GSN scores ranged from 1.38 to 1.72 from youngest to oldest females, and from 3.23 to 3.53 from youngest to oldest males (Table 2). For females, the difference among age groups was significant (ANOVA *F* = 7.82, *p* = 0.0005), but not for males (ANOVA *F* = 2.05, *p* = 0.1303). The Pearson chi-squared test revealed similar results when the score distribution was assessed for each sex by age group: There was a significant difference in the score distribution among age groups in females (Pearson chi^2^ = 17.8256, *p* = 0.001), but not in males (Pearson chi^2^ = 5.7630, *p* = 0.674). Figure 3 shows the age-specific GSN score distribution in Australian males and females.

**Table 2:** Mean GSN scores by sex and age group in the Australian sample

Age group	Male		Female	
	*N*	Mean (SD)	*N*	Mean (SD)
18–49 years	131	3.23 (0.91)	120	1.38 (0.50)
50–69 years	123	3.35 (0.97)	71	1.58 (0.67)
70 + years	55	3.53 (0.88)	67	1.72 (0.62)

When individual age groups were compared, a significant difference in the GSN score distribution was found between females aged 18–49 years and the other two groups aged 50–69 years and 70 + years (Pearson chi^2^ = 9.6840, *p* = 0.008, and Pearson chi^2^ = 16.2262, *p* < 0.0001, respectively). There was no significant difference between 50–69 years and 70 + year-old females (Pearson chi^2^ = 3.5723, *p* = 0.168). There was also no significant difference between any of the male age groups: 18–49 years v. 50–69 years (Pearson chi^2^ = 1.6525, *p* = 0.799), 18–49 years vs. 70 + years (Pearson chi^2^ = 4.8341, *p* = 0.305), and 50–69 years vs. 70 + years (Pearson chi^2^ = 2.8988, *p* = 0.575).

Logistic regression analysis was performed using the total sample—first without age—to assess the contribution of GSN score to the sex estimation model (Table 3). The results indicate that the GSN score significantly (*p* < 0.001) contributes to the model, and the model explained 57.7% of the variance in the sex estimate (pseudo R^2^). The cross-validated accuracy of the model was 87.5% (81.6% for males, 94.6% for females) with a sex bias of − 13.0% (Table 4). Next, age was added to the model as a confounding variable (Table 3), resulting in the same cross-validated accuracy of 87.5% with a slightly improved sex bias of − 11.4% (Table 4). Age significantly contributed to the model (*p* = 0.003), and the pseudo-R^2^ was 58.9%, meaning that the model with age predicts sex slightly better than the model without age as a confounding variable.

**Table 3:** Overall and age-group-specific logistic regression formulae and associated statistics for sex estimation (0 = male, 1 = female) in the Australian sample

	Logistic regression (LR) formula (sex =)	95% CI	LR	LR
		of GSN	*p*-value	Pseudo-R^2^
Total sample without age	− 2.930*GSN + 6.552	− 3.42	< 0.0001	0.5772
		to		
		− 2.441		
Total sample with age	− 3.029*GSN + 0.021*age + 5.679	− 3.535	< 0.0001	0.5892
		to		
		− 2.523		
Total sample with age group	− 3.029*GSN + 0.051*age group 2 + 1.344*age group 3 + 6.448	− 3.54	< 0.0001	0.5959
		to		
		− 2.518		
18–49 years	− 3.824*GSN + 8.078	− 4.965	< 0.0001	0.6562
		to		
		− 2.682		
50–69 years (age group 2)	− 2.446*GSN + 5.226	− 3.128	< 0.0001	0.5111
		to		
		− 1.764		
70 + years (age group 3)	− 3.079*GSN + 7.915	− 4.139	< 0.0001	0.6016
		to		
		− 2.019

**Table 4:** Overall and sex-specific cross-validated classification accuracy and sex bias values derived from the logistic regression formulae in Table 3

	Cross-validated accuracy Overall	Cross-validated accuracy Males	Cross-validated accuracy Females	Sex bias
Total sample without age	87.5%	81.6%	94.6%	− 13.0%
Total sample with age	87.5%	80.3%	91.7%	− 11.4%
18–49 years	88.8%	79.4%	99.2%	− 19.8%
50–69 years	84.5%	81.3%	90.1%	− 8.8%
70 + years	89.3%	87.3%	91.0%	− 3.7%

Furthermore, models were calculated for the individual age groups as shown in Table 3. The cross-validated accuracies and sex bias values for these models are listed in Table 4. The worst performing model is the one for the 50- to 69-year-age group, with a pseudo-*R*^2^ of 51.1% and classification accuracy of 84.5%. The model for the 70 + years age group was most accurate relative to male (87.3%) and overall classification accuracy (89.3%), with an acceptable sex bias of − 3.7%. However, this was to the detriment of female classification accuracy, which was 91% versus 99.1% in the youngest (18–49 years) age group. The prediction ability of the model for the 70 + years age group was the second highest overall at 60.2%; the highest (pseudo-*R*^2^ = 65.6%) was in the model for the 18- to 49-year-age group.

## Discussion

The effect of age has not been widely studied relative to biological covariance. As populations age, it is important to understand the influence of aging on the skeleton. Walker and a handful of other publications concerning morphoscopic analysis suggest no significant association between sciatic notch shape and age but instead a likely association between population and secular changes that are related to population specificity [24, 25]. However, morphometric and GMM studies by Biwasaka et al. [8] and [10] suggest otherwise. Therefore, understanding the extent to which age affects sex estimation of the GSN morphology is important toward providing a biological profile for unidentified human remains.

Intra-observer agreement results indicate that the Walker [42] method can be scored reliably on MDCT scans. Furthermore, the intra-observer results from this study were comparable to those of Colman et al. [12], investigating the accuracy of 3D virtual bone models of the pelvis for morphological sex estimation. Additionally, there was no significant bilateral asymmetry, a finding that supports previous research [16, 37].

The aim of this study was to explore the effect of age on the distribution of GSN scores according to the Walker [42] method. Overall, the female distribution of the Australian populations reflects the original article, with the highest proportion having a score of one, although the value was not as high as in the original study (53% versus 65%). However, none of the Australian females received a score within the male range compared to Walker’s study, where 2% of females were scored 4 or 5. The male distribution assessment of the GSN differs from Walker’s original study, whereby 43% of males were assigned a 2, compared to 17% in the present study. As in Walker’s study, males were assigned the whole spectrum of scores from 1 to 5.

In the Australian sample, the mean scores increased with age both for males and for females. However, the increase was only statistically significant in females. By comparing GSN score distribution between the individual age groups, it was demonstrated that the significant difference in females is related to the distribution in older females being shifted toward higher scores compared to the premenopausal age group of 18–49 years. This suggests that older females present narrower GSN morphology.

These findings accord with the few studies available in the published literature. For example, Biwasaka et al. [8] quantitatively analyzed sex and age-dependent morphological features of the coxal bone, applying geometric morphometrics (GMM) to three-dimensional CT scans of pelvic bones of a contemporary Japanese population aged 16 to 100 years. They report that the GSN of females deepened with age and overall sex estimation accuracy rates as high as 93% (males, 93.1%; females, 94.8%) using average corresponding point differences from the GMM models. Recently, Byram et al. (2022) quantified GSN shape relative to age, sex, and ancestry using elliptical Fourier analysis. They reported that sex has the most significant effect on shape, with older females displaying GSN morphology similar to males with increasing age.

In the present study, age significantly contributed to the sex estimation prediction model, and some age-group-specific models achieved higher sex classification accuracy (18–49 years, 88.8%; 70 + years, 89.3%) than the total sample model (87.5%). The cross-validated classification accuracy for the Australian-specific models was 87.5%, which is higher than the accuracy (80.0%) when all specimens have been classified as reported in the original Walker paper. The sex bias for the Australian sample was − 13%, which indicates proportionately lower classification accuracy in males compared to the 27.8% sex bias of the Walker method.

This study empirically demonstrated the influence of age on GSN score distribution following the Walker [42] method. Given the significant increase in mean scores with age in females, the female classification accuracy of age-specific models decreased with age. Irrespective of the latter, the most accurate model (89.3% classification accuracy) was for the 70 + -year-old Australian group, with males achieving a slightly lower classification accuracy (87.3%) than did females (91%), resulting in an acceptable sex bias of 3.7%. The cross-validated classification accuracy in all the models above 85% suggests that these may be applicable in forensic sex estimation in Australia and possibly beyond.

## Conclusion

The present study demonstrated that age affects GSN morphology using the scoring system of Walker [42]. Higher mean scores in older females imply that GSN becomes, on average, narrower with increasing age, which has been assumed to be related to the effect of age-related hormonal changes on bone metabolism. It is recommended that forensic practicioners consider estimated age when assessing sex based on GSN morphology in unidentified human remains. Further research regarding the specific effect of demographic and health variables on sex estimation would be beneficial to improve prediction accuracy of this biological attribute.
